# Nurr1 deficiency impairs autophagy-lysosomal function through GBA-dependent transcriptional regulation in Parkinson’s disease pathogenesis

**DOI:** 10.3389/fnagi.2025.1612389

**Published:** 2025-06-30

**Authors:** Cheng Cheng, Huijia Yang, Lulu Tian, Yang Ni, Congcong Jia, Weidong Le, Qingshan Wang

**Affiliations:** ^1^School of Public Health, Dalian Medical University, Dalian, China; ^2^Liaoning Provincial Key Laboratory for Research on the Pathogenic Mechanisms of Neurological Diseases, The First Affiliated Hospital, Dalian Medical University, Dalian, China; ^3^Department of Neuroelectrophysiology, The First Affiliated Hospital of USTC, University of Science and Technology of China, Hefei, China; ^4^Institute of Brain Science and Brain-Inspired Research, Shandong First Medical University and Shandong Academy of Medical Sciences, Jinan, China

**Keywords:** Parkinson’s disease, Nurr1, the autophagy-lysosomal pathway dysfunction, lysosomal impairment, GBA

## Abstract

**Introduction:**

The autophagy-lysosomal pathway (ALP) dysfunction and lysosomal impairment contribute to the pathogenesis of Parkinson’s disease (PD). Nuclear receptor related protein 1 (Nurr1) maintains the differentiation and maturation of dopaminergic neurons, and mutants or polymorphism in Nurr1 is associated with familial and sporadic PD. Previous studies on Nurr1 have mainly focused on the development and maintenance of midbrain dopaminergic neurons, while the potential involvement of Nurr1 in ALP regulation remains uncharacterized.

**Methods:**

Stable Nurr1 knockdown cells and inducible Nurr1 knockout mice were generated. Transcriptome sequencing and analysis was utilized to confirm the altered pathways and differentially expression genes associated with ALP. Transmission electron microscopy observation was conducted to find the ultrastructure differences between the Nurr1 knockdown cells and the controls. The expression of LC3B and the colocalization of LC3B and Lamp1 were assessed. Lysosomal acidity in the Nurr1 knockdown cells and the controls was measured. The expression of lysosomal proteins (Lamp 1/2, CTSD, and GBA) was determined *in vitro* and *in vivo* in the Nurr1-deficient models. Dual-luciferase reporter gene assay was performed to detect the transcriptional activity of GBA. The key lysosomal proteins (Lamp 1/2 and CTSD) were assessed after GBA overexpression.

**Results:**

Twenty-two terms and 45 differentially expression genes associated with ALP were identified by transcriptome analysis. Knockdown of Nurr1 induced intracellular aggregation of autophagosomes, increased endogenous expression of LC3B II and elevated colocalization of exogenous GFP-LC3B with Lamp1. Lysosome dysfunction has been implicated with lysosomal alkalization and deprived level of lysosomal marker proteins with Nurr1 deficiency. GBA was transcriptionally downregulated by Nurr1 and Nurr1 deficiency-triggered lysosomal dysfunction were attenuated by GBA overexpression in the Nurr1 knockdown cells.

**Discussion:**

Our study provided the first experimental evidence that Nurr1 deficiency induced lysosomal dysfunction by alkalizing the lumen of lysosomes and downregulating the key lysosomal protein (Lamp1, CTSD, and Lamp2) expression *in vivo* and *in vitro*. Defective lysosomal function compromised lysosomal mediated autophagic vesicle clearance. Mechanistically, Nurr1 transcriptionally regulated GBA expression, which in turn governed lysosomal marker protein homeostasis through a GBA-dependent axis. This study illuminated the involvement of Nurr1 in the ALP and the interaction between PD-related genes in the pathogenesis of PD.

## Introduction

Parkinson’s disease (PD) is a progressive neurodegenerative disease characterized by the degeneration of dopaminergic (DAergic) neurons in the substantia nigra (SN) and the accumulation of misfolded proteins within susceptible neurons (Han and Le, 2023). Nuclear receptor related protein 1 (Nurr1), an orphan nuclear receptor, is widely involved in various signaling pathways associated with human diseases, particularly neurological and neurodegenerative diseases (Chen et al., 2020). As a transcription factor, Nurr1 regulates the expression of tyrosine hydroxylase (TH), aromatic amino acid decarboxylase (AADC), and the dopamine transporter (DAT) which are essential for the synthesis, storage, and release of dopamine (DA) (Jankovic et al., 2005; Chen et al., 2018), indicating that Nurr1 has a vital role in maintaining the differentiation and maturation of DAergic neurons (Al-Nusaif et al., 2022). Homozygous Nurr1-deficient (*Nurr1*^–/–^) mice die shortly after birth due to the complete absence of DAergic neurons in the SN and ventral tegmental area (Zetterström et al., 1997). 1-Methyl-4-phenyl-1,2,3,6-tetrahydropyridine (MPTP) is used to simulate PD conditions in animal models (Wu et al., 2022) and lower Nurr1 expression increases the mesencephalic DAergic neurons vulnerability to MPTP in *Nurr1*^+/–^ mice (Le et al., 1999). Downregulated expression of Nurr1 has also been detected in the postmortem brains of sporadic PD (Chu et al., 2006; Moran et al., 2007), particularly in the SN DAergic neurons with α-syn inclusions (Chu et al., 2006). Moreover, Nurr1 agonists reverse the behavioral and histological abnormalities in animal models of PD (Kim et al., 2015).

In mammals, the autophagy-lysosomal pathway (ALP) is one of the principal systems for misfolded protein degradation (Du et al., 2009; Li Y. et al., 2023). Previous studies indicate that ALP dysfunction plays a critical role in both familial and sporadic forms of PD (Beilina et al., 2014; Moors et al., 2018). The lysosome is a multifactorial organelle containing various proteases (e.g., cathepsins and hydrolases) which digests misfolded proteins and damaged organelles, serving as the final step of the ALP. Impaired lysosomal function results in the accumulation and aggregation of abnormal proteins and toxic waste, ultimately leading to synaptic loss and neuronal death in age-related neurodegenerative diseases (Pan et al., 2008; Hossain et al., 2020). Mounting evidence has revealed a causal link between lysosomal impairment and PD (Dehay et al., 2013). ALP/lysosomal markers, including microtubule-associated protein 1 light chain 3 (LC3), lysosomal-associated membrane protein 1/2 (Lamp1 and Lamp2), cathepsin-D (CTSD), and glucocerebrosidase (Gcase), have been identified as components of the Lewy bodies (LBs) in patients with sporadic PD (Dehay et al., 2013). LBs may seed around impaired lysosomes or undegraded autophagosomes and grow in size with the deposition of lysosomal/AP-derived undegraded material as the disease progresses (Dehay et al., 2013).

The GBA gene encodes the lysosomal enzyme β-glucocerebrosidase (GCase), catalyzing the hydrolysis of glucosylceramide (GlcCer) into ceramide and glucose (Pradas and Martinez-Vicente, 2023). As a lysosomal protein, GCase is synthetized in the endoplasmic reticulum (ER) and subsequently transported to lysosomes to exert its function (Blanz et al., 2015), indicating that GBA dysfunction is associated with general lysosomal dysfunction and disruption of autophagy (Gan-Or et al., 2015). Homozygous mutations in the GBA gene cause Gaucher’s disease (GD), an inherited autosomal recessive lysosomal storage disorders (LSDs) (Bourdenx et al., 2016), which also significantly increases the risk of PD. Among all PD-related genes, GBA is currently considered the most common genetic risk factor, with a mutation frequency of 5%–15% (Smith and Schapira, 2022). PD patients exhibit reduced GCase activity across blood, cerebrospinal fluid (CSF) and brain tissues (Senkevich and Gan-Or, 2020). Loss of GBA function leads to extensive lysosomal dysfunction by altering intralysosomal pH, impairing lysosomal membrane stability and attenuating the activity of other lysosomal enzymes (Navarro-Romero et al., 2022).

Previous studies on Nurr1 have mainly focused on the development and maintenance of midbrain DAergic neurons (Jankovic et al., 2005; Kadkhodaei et al., 2013). Nurr1 regulates the expression of genes associated with mitochondrial function and oxidative phosphorylation (Puigserver and Spiegelman, 2003), and protects DAergic neurons from inflammation by suppressing the expression of inflammatory genes (Dong et al., 2020; Li T. et al., 2023). While the potential involvement of Nurr1 in ALP regulation remains uncharacterized, our study provides the first experimental evidence that Nurr1 deficiency induces lysosomal dysfunction and compromises lysosomal mediated autophagic vesicle clearance. Mechanistically, Nurr1 transcriptionally regulates GBA expression, which in turn governs lysosomal marker protein homeostasis through a GBA-dependent axis.

## Materials and methods

### Cell culture and transfection

Mouse neuroblastoma N2a cells were cultured in Dulbecco’s modified Eagle’s medium (DMEM, Meilunbio, Dalian, China) supplemented with 10% fetal bovine serum (FBS, Gibco, MA, USA) and 1% penicillin/streptomycin solution (Meilunbio) in 5% CO_2_ at 37°C. N2a cells were transfected with pLKO-puro-shRNA-Nurr1 (Nurr1-KD) or pLKO-puro-shRNA (Ctrl) plasmid by lipofectamine 6,000 reagents (Beyotime, Shanghai, China) according to the manufacturer’s instructions, respectively. Transfected cells were screened by cell culture medium containing 3 μg/ml puromycin (Beyotime) until no more cell died.

For exogenous LC3B detection, the Nurr1-KD and Ctrl cells were transfected with GFP-LC3B plasmids by lipofectamine 6,000 reagents for 48 h. After that, cells were digested and seeded onto glass coverslips pre-coated with poly-lysine in 24 well-plate dishes and cultured overnight. The above cells were stained with an anti-LAMP1 antibody (methods were mentioned in the immunofluorescence staining and imaging part). For GBA overexpression and transcriptional activity assay, the coding domain sequence (NM_001077411.4) or its upstream 2,000 bp promoter sequence of mouse GBA was subcloned into pEGFP-N1 (GBA-pEGFP-N1) or pGL4.18 (GBA2000-luc) plasmid, respectively. GBA-pEGFP-N1/pEGFP-N1 plasmid was transfected into Nurr1-KD or Ctrl cells for 72 h. GBA2000-luc/pGL4.18 (Ctrl-luc) and pGL4.74 plasmids were co-transfected into Nurr1-KD and Ctrl cells for 48 h.

### Generation of inducible Nurr1 knockout mice

Nurr1*^floxp/floxp^* mice were generated by ViewSolid Biotech (Beijing, China) and bred with CAGGCre^*ER*TM^ (Cre) mice. Cre-Nurr1^floxp/wt^ mice were used to cross with Nurr1^floxp/floxp^ mice to obtain the Cre-Nurr1^floxp/floxp^ mice (Nurr1^cKO^). The Nurr1^floxp/floxp^ mice (Nurr1^cWT^) were used as control. The genotype of mice was identified by PCR screening using primers as follows: Nurr1-floxp-forward: 5′-gggggcgtttggaaatga-3, Nurr1-floxp-reverse: 5′-gggcaaaagaggtggaacagc-3′; cre-forward: 5′-gctaaccatgttcatgccttc-3′, cre-reverse: 5′-aggcaaattttggtgtacgg-3′; positive control-forward: 5′-gagtgccaattcgatgatgagtc-3′, positive control-reverse: 5′-gcgcttactttgtgctgtccta-3′. Eight-week-old Nurr1^cKO^ and Nurr1^cWT^ mice were injected intraperitoneally with 10 mg/ml tamoxifen twice daily for 5 consecutive days. After 1 month, Nurr1^cKO^ and Nurr1^cWT^ mice were used (*n* ≥ 4 for each group) for further analysis.

### Real-time quantitative PCR

Total RNA was extracted from N2a cells using RNAiso Plus reagent (Takara, Japan) and reverse-transcribed to double-strand cDNA according to the manufacturer’s instructions (TransGene Biotech, Beijing, China). The relative gene expression was determined with a SYBR Green RT-qPCR kit (TransGene Biotech) by ABI Prism 7,500 Detection System (Applied Biosystems, Foster City, CA, USA). Real-time quantitative PCR (RT-qPCR) assay was repeated three times with three replicates per detection and gene expression was quantified with the 2^ΔΔCt^ method. The primers sequences were as follows: Nurr1 (forward: 5′-attccaggttccaggcaaac-3′, reverse: 5′-agcaaagccagggatcttct-3), GAPDH (forward: 5′-gcattgtggaagggctcatg-3′, reverse: 5′-agggatgatgttctgggcag-3′), GBA (forward: 5′-catccttgctttgtccccac-3′, reverse: 5′-ctggaagtcgttaggggtgt-3′).

### Western blotting assay

Cells and SN tissues were re-suspended in radio immunoprecipitation assay (RIPA) buffer (Beyotime) with 1% protease inhibitor cocktail (Sigma-Aldrich, St. Louis, MO, USA) and incubated on ice for 30 min, then centrifuged at 12,000 rpm for 15 min. The concentration of the supernatant was determined by a BCA Protein Assay kit (Takara). The remaining supernatant was added 5× sodium dodecyl sulfate (SDS) loading buffer (Beyotime) and boiled at 95°C for 10 min. Samples with equal amount of protein (20–30 μg) were subjected to 10% or 12.5% SDS-polyacrylamide gel electrophoresis (PAGE) and then transferred to 0.2/0.45 μm polyvinylidene difluoride (PVDF) membranes (Merck Millipore Ltd., Germany). After blocking with 5% skimmed milk for 1 h at room temperature (RT), the membranes were incubated with the primary antibodies at 4°C overnight. After incubation, the membranes were washed with 1× tris-buffered saline containing tween-20 (TBST) three times and incubated with horseradish peroxidase (HRP)-conjugated secondary antibodies at RT for 1 h. Specific protein bands were developed with enhanced electrochemiluminescence (ECL) plus procedure (Meilunbio) and quantified by the FluorChem Q system (Protein Simple, San Jose, CA, USA), normalized to GAPDH. Cell samples were collected at least three times, and the assays were repeated three times regarding each sample. Antibodies used were as follows: anti-Nurr1/NR4A2 antibody, anti-Cathepsin D antibody (immunofluorescence staining, IF), anti-CD107a/LAMP1 (1D4B) antibody (IF), anti-CD107b/LAMP2 antibody (IF), anti-LC3 antibody (Proteintech Group Inc., Rosemont, IL, USA), anti-β-glucosidase (GBA) antibody (Santa Cruz Biotechnology, TX, USA), anti-Cathepsin D antibody (Western blotting, WB), anti-LAMP1 antibody (WB), anti-LAMP2A antibody (WB) (Abcam, Cambridge, MA, USA), anti-GAPDH antibody (Cell Signaling Technology, Danvers, IL, USA), HRP-conjugated secondary antibodies (Proteintech). The original images of Western Blotting are available in the Supplementary material.

### Immunofluorescence staining and lysosomal acidity assay

Cells were washed with phosphate buffered saline (PBS, Meilunbio) after seeding for 24 h and fixed in 4% paraformaldehyde (PFA, Beyotime) for 30 min at RT, then permeated with 0.25% Triton X-100 (Sigma-Aldrich) for 30 min at RT. Cell samples were washed with PBS and blocked with 5% bovine serum albumin (BSA, Sigma-Aldrich) for 30 min at RT. The samples were stained with appropriate primary antibodies overnight at 4°C. After staining, the samples were washed and incubated with fluorescent secondary antibodies (Cell Signaling Technology) in a dark place for 1 h at RT. Nuclei were stained with Hoechst (1:1000) for 5 min. The fluorescence images were taken under a confocal microscope (A1R MP+, Nikon, Japan). Brain tissues were fixed with 4% PFA overnight at 4°C, then dehydrated in 15% and 30% sucrose solutions for 24 h and embedded in an optimal cutting temperature compound (Tissue-Tek, Torrance, CA, USA). Coronal sections of 40 μm were cut on a freezing microtome (Leica, Wetzlar, Germany). The slides were blocked with buffer (10% normal goat serum, 1% BSA, 0.3% Triton X-100, PBS solution) for 1 h at RT and incubated with the primary antibodies overnight at 4°C. Following incubation with the primary antibody, the slides were washed with PBS and incubated with secondary antibodies. Nuclei were stained with Hoechst (1:1000) for 5 min. The fluorescence images were taken under a confocal microscope (A1R MP+, Nikon, Japan).

Cells were seeded into a 6-well plate at a density of 5 × 10^5^ per well for 16 h. Then the samples were washed with PBS and incubated with 2 μM LysoSensor Green DND-189 dye (TransGene) at 37°C for 20 min. Fresh DMEM with 10% FBS was added to the plates after washing with PBS for three times. Then the samples were photographed by a confocal microscope (Nikon) or harvested and quantified by a flow cytometer (FACS CANTO II, BD, USA) at the wavelength of 530 nm. Cells without being stained were used as negative control.

### Dual-luciferase reporter gene assay

Cells were harvested in a lysis buffer after transfection for 48 h and then centrifuged at 12,000 rpm for 10 min to get the supernatant. The activities of the firefly and renilla luminescence were measured by a dual-luciferase assay kit (Beyotime) under a microplate reader (BioTek Instruments, Inc., VT, USA). The concentration of the supernatant was determined with a BCA protein kit (Takara). The relative luciferase activity was normalized to the renilla activity and protein concentration.

### Transcriptome sequencing

The transcriptome sequencing and analysis were conducted by OE biotech Co., Ltd. (Shanghai, China). In brief, total RNA of Nurr1-KD and Ctrl cells was extracted by the RNAiso Plus reagent (Takara) and evaluated. The libraries were constructed by TruSeq Stranded mRNA LT Sample Prep Kit according to the manufacturer’s instruction (Illumina, San Diego, CA, USA) and then sequenced on the Illumina sequencing platform. DAVID online platform was utilized to perform the Gene Ontology (GO) enrichment analysis to identify significant canonical pathways. The differentially expressed genes (DEGs) were screened out with the DESeq2 Software. Gene expression was estimated by basemean value and significant difference was tested by negative binomial (NB) distribution method. Fold change ≥ 1.5 and false discovery rate (*q*) < 0.05 were set as the cut-off criteria for identifying the DEGs.

### Transmission electron microscopy observation

Cells were seeded at a density of 6 × 10^6^ in a 10 cm dish and cultured at 37°C overnight. On the next day, the cells were harvested and washed with PBS. Then the cells were pre-fixed with a fixative solution containing 2.5% glutaraldehyde (Servicebio, Wuhan, China) at RT for 30 min, and post-fixed in 1% osmium acid at RT for 2 h, successively dehydrated with graded ethanol (30%–50%–70%–80%–90%–95%–100%–100%). After embedding steps, the samples were double stained with 2% uranyl acetate saturated alcohol and 2.6% lead citrate solution, placed on grid and imaged by a transmission electron microscopy (TEM) (HITACHI, HT7700).

### Enzyme activity assay of GBA

Cells were seeded at a density of 6 × 10^6^ into a 10 cm dish and cultured at 37°C overnight. On the next day, cells were sonicated in PBS and then centrifuged at 12,000 rpm for 15 min to get the supernatant. The supernatant was mixed with the substrate of Gcase and a compatible buffer from a β-Glucosidase enzyme activity kit (YanQi Biology, Shanghai, China) at 37°C for 2 h, then added the chromogenic reagents. The protein concentration of the supernatant was determined by a BCA kit (Takara). The standard curve was established with the concentration of the standard substance and its absorbance at 405 nm on a microplate reader (BioTek Instruments). The generation of 1 nmol p-nitrophenol from every milligram of protein per minute was defined as a unit of enzyme activity (nmol/min/mg protein).

### High-performance liquid chromatography

The SN was rapidly removed, weighed and kept at −80°C. The amounts of DA, 3,4-dihydroxyphenylacetic acid (DOPAC), homovanillic acid (HVA), 3-methoxytyramine (3-MT) and 5-hydroxytryptamine (5-HT) were determined by high-performance liquid chromatography (HPLC) (EiCOM, HTEC-500, Kyoto, Japan) as described in detail previously (Xiao et al., 2015).

### Statistical analysis

GraphPad Prism 8.0 software was used for statistical analysis and all data were expressed as mean ± SEM. Unpaired Student’s *t*-test was used for comparisons between two groups. Statistical significance was defined as *p*-value less than 0.05, *p < 0.05; **p < 0.01; ***p < 0.001; ****p < 0.0001.

## Results

### Verification of the stable Nurr1 knockdown cells

**FIGURE 1:**
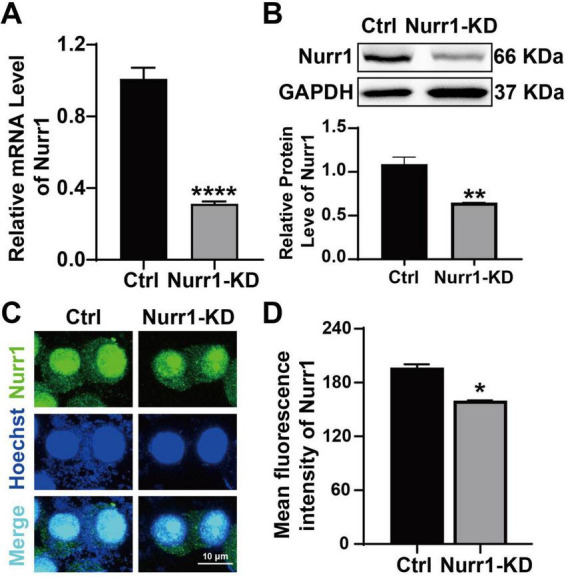
Verification of stable Nurr1 knockdown cells. **(A)** The mRNA level of Nurr1 was detected in Ctrl and Nurr1-KD cells by RT-qPCR. **(B)** The protein expression of Nurr1 was indicated by Western blotting in both groups. **(C)** Immunofluorescence staining was used to confirm the expression of Nurr1 (green) in both groups, with nuclei counterstained using Hoechst (blue). **(D)** The mean fluorescence intensity of Nurr1 was quantified. Data were expressed as mean ± SEM. *p < 0.05 vs. Ctrl cells, **p < 0.01 vs. Ctrl cells, ****p < 0.0001 vs. Ctrl cells. Cell samples were collected three times with each detection repeated three times.

We established stable Nurr1 knockdown (Nurr1-KD) and control (Ctrl) cells. The expression of Nurr1 was evaluated by RT-qPCR, Western blotting and Immunofluorescence staining. The results revealed that the mRNA and protein levels of Nurr1 in the Nurr1-KD group were significantly decreased to 31% (*p* < 0.0001) and 64% (*p* < 0.01) of those in the Ctrl group, respectively (Figures 1A, B). Consistently, immunofluorescence staining showed markedly weaker staining of Nurr1 intensity in the Nurr1-KD group compared to the Ctrl group (*p* < 0.05, Figures 1C, D), in accordance with the results of RT-qPCR and Western blotting. These results confirm the successful construction of Nurr1 knockdown cells.

### Nurr1 participates in the ALP predicted by transcriptome analysis

**FIGURE 2:**
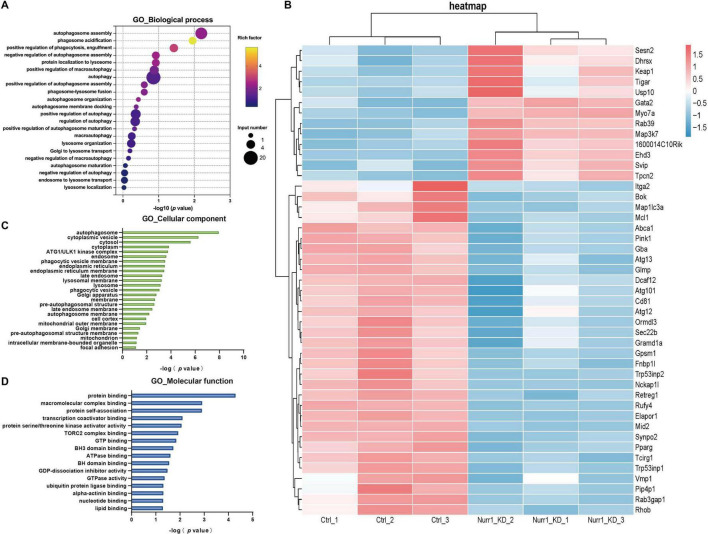
Transcriptome analysis reveals ALP-related alterations in Nurr1-KD vs. Ctrl cells. **(A)** Gene Ontology (GO) enrichment analysis of biological processes (BP) associated with the autophagy-lysosome pathway (ALP), performed by the DAVID bioinformatics platform. **(B)** Heatmap visualization of 45 differentially expressed genes (DEGs) (fold change ≥1.5, q < 0.05) annotated to ALP-related biological processes, identified via DESeq2 differential expression analysis. GO enrichment analysis by cellular component **(C)** and molecular function **(D)**. Cells were collected with three replicates for transcriptome sequencing.

Transcriptome analysis identified 1,785 DEGs between Nurr1-KD and Ctrl groups, including 835 upregulated genes and 950 downregulated genes (Fold change ≥ 1.5, *q* < 0.05). GO identified 22 biological pathways associated with ALP (Figure 2A), containing 45 DEGs with 13 upregulated and 32 downregulated genes (Figure 2B). Further analysis showed that these DEGs were mainly enriched in the following categories: autophagy assembly, phagosome acidification, positive regulation of phagocytosis/engulfment, etc. by biological process (Figure 2A); autophagosome and cytoplasmic vesicle, etc. by cellular component (Figure 2C); protein binding and macromolecular complex binding, etc. by molecular function (MF) (Figure 2D). Collectively, all these analysis results demonstrate a strong association between Nurr1 and the ALP.

### Knockdown of Nurr1 increases the number of autophagic vesicles and LC3B II expression level

**FIGURE 3:**
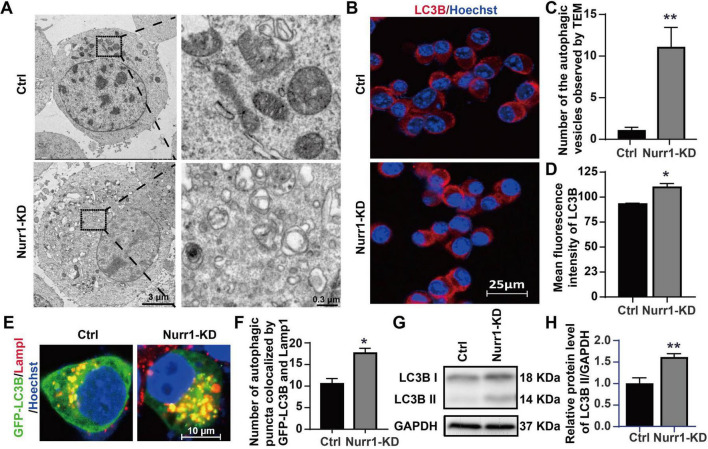
Changes in autophagosomes by TEM observation and detection of LC3B. **(A)** Ultrastructure differences showed by TEM observation. The enlarged part of Nurr1-KD cells indicated the autophagic vesicles. **(B)** Immunofluorescence staining of endogenous LC3B (red), with nuclei counterstained using Hoechst (blue). **(C)** The number of the autophagic vesicles observed by TEM was quantified. **(D)** The mean fluorescence intensity of LC3B was quantified. **(E)** Colocalization of transfected exogenous LC3B (green) and Lamp1 (red) indicated the autophagic puncta (yellow). **(F)** The number of autophagic puncta colocalized by GFP-LC3B and Lamp1 was quantified. **(G)** Western blotting detection of endogenous LC3B. **(H)** The protein level of LC3B was normalized by GAPDH and quantified. Data were expressed as mean ± SEM. *p < 0.05, **p < 0.01 vs. Ctrl cells. Samples were collected at least three times with three replicates.

TEM observation was performed to find the ultrastructure differences between Nurr1-KD and Ctrl group. A sharply increased number of autophagic vesicles were observed in Nurr1-KD group (*p* < 0.01, Figures 3A, C), indicating a dysregulated autophagic activity. Immunofluorescence analysis exhibited an increased LC3B fluorescence intensity and more LC3B-positive punctate structures in Nurr1-KD cells compared to Ctrl cells (*p* < 0.05, Figures 3B, D). Then, we transfected GFP-LC3B plasmids into Nurr1-KD and Ctrl cells. Immunofluorescence analysis showed that knockdown of Nurr1 significantly increased the autophagic puncta colocalized by exogenous LC3B and Lamp1 (*p* < 0.05, Figures 3E, F). Western blotting assay suggested the enhanced conversion of LC3B-I to LC3B-II, indicating elevated number of the autophagosomes (*p* < 0.01, Figures 3G, H). These results indicate that knockdown of Nurr1 promotes autophagosome accumulation.

### Knockdown of Nurr1 disrupts the lysosomal acidity and reduces the lysosomal marker proteins expression

**FIGURE 4:**
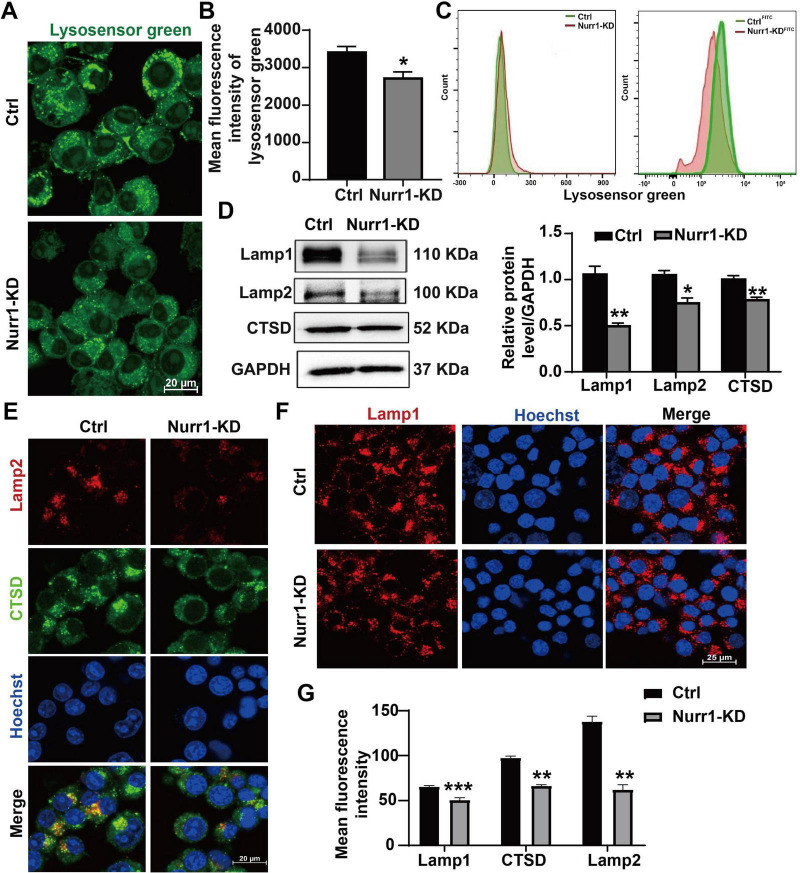
Effects of Nurr1 knockdown on lysosomal acidity and lysosomal protein expression *in vitro*. **(A)** Cells were stained by LysoSensor Green DND-189 and observed by a confocal microscopy and **(B)** the mean fluorescence intensity was determined by flow cytometry analysis. **(C)** Images of flow cytometry indicated cells without/with LysoSensor Green, respectively. **(D)** Western blotting results of representative lysosomal markers (Lamp1, Lamp2, and CTSD). The protein levels were normalized by GAPDH. **(E)** Co-staining of CTSD (green) and Lamp2 (red) indicated the CTSD expression in the lysosomes. **(F)** Immunofluorescence staining of Lamp1 (red). **(G)** Quantification of the fluorescence intensity of lysosomal proteins in Nurr1-KD cells and Ctrl. Data were expressed as mean ± SEM. *p < 0.05 vs. Ctrl cells, **p < 0.01 vs. Ctrl cells, ***p < 0.001 vs. Ctrl cells. Samples were collected five times with three replicates.

Lysosomal acidity in Nurr1-KD and Ctrl cells was measured by the LysoSensor Green DND-189, which showed a pH-dependent increase in green fluorescence intensity in an acidified environment. The fluorescence intensity in Nurr1-KD cells showed a significant decrease compared to Ctrl cells (Figure 4A), indicating that knockdown of Nurr1 induced the alkalization of lysosomes. The same results were further confirmed by flow cytometry analysis (*p* < 0.01, Figures 4B, C). The expression of key lysosomal proteins (Lamp1, Lamp2, and CTSD) was assessed using Western blotting and Immunofluorescence staining. The results showed that the protein levels of Lamp1, Lamp2, and CTSD were all decreased to 46.8% (*p* < 0.01), 70.9% (*p* < 0.05), and 77.6% (*p* < 0.01), respectively (Figure 4D). A consistent alteration pattern in the expression of Lamp1, Lamp2, and CTSD was observed in Nurr1-KD cells through Immunofluorescence staining analysis (*p* < 0.01, Figures 4E–G). Immunofluorescence quantification further revealed diminished lysosomal localization of CTSD in Nurr1-KD cells (*p* < 0.01, Figures 4E, G), indicating impaired lysosomal protease retention. The above results demonstrate that knockdown of Nurr1 disrupts normal lysosomal function.

### Inducible Nurr1 knockout mice demonstrate decreased dopamine metabolite level and reduced expression of lysosomal proteins

**FIGURE 5:**
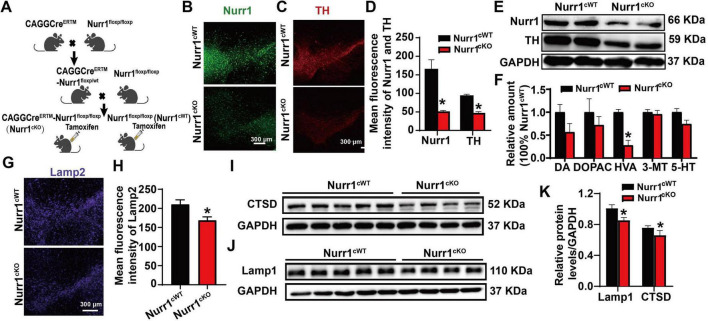
Lysosomal protein change in the inducible Nurr1 knockout mice. **(A)** Generation of the inducible Nurr1 knockout mice. **(B)** The expression of Nurr1 in the SN determined by immunofluorescence staining. **(C)** The expression of TH in the SN detected by immunofluorescence staining. **(D)** Quantification of the mean fluorescence intensity of TH and Nurr1 in the SN. **(E)** Western blotting showed the expression of Nurr1 and TH in the SN. **(F)** The amount of DA and its metabolites in the SN detected by HPLC. **(G)** The expression of Lamp2 in the SN indicated by immunofluorescence staining. **(H)** Quantification of the mean fluorescence intensity of Lamp2. **(I,J)** The expression of Lamp1/CTSD was detected by Western blotting. **(K)** Relative quantification of the expression of Lamp1 and CTSD, normalized by GAPDH. Data were expressed as mean ± SEM. *p < 0.05 vs. the Nurr1^cKO^ mice. The number of mice in each group was ≥4.

To further verify the regulation of Nurr1 in lysosomal function, we have established an inducible Nurr1 knockout mouse model (Nurr1^cKO^) (Figure 5A) and systematically assessed the PD associated parameters. Immunofluorescence and Western blotting analysis confirmed efficient Nurr1 protein reduction in the SN (*p* < 0.05, Figures 5B, D, E), accompanied by a corresponding decline in TH expression (*p* < 0.05, Figures 5C–E). HPLC revealed significant depletion of HVA in Nurr1^cKO^ mice (*p* < 0.05), with non-significant decreasing trends in DA, DOPAC, and 5-HT levels. Notably, 3-MT concentrations remained unaltered (Figure 5F). Strikingly, lysosomal marker proteins Lamp1, Lamp2, and CTSD were significantly downregulated in the SN of Nurr1^cKO^ mice (*p* < 0.05; Figures 5G–K), mirroring our *in vitro* findings (Figures 4D–G). These congruent results establish Nurr1 as a key transcriptional regulator of lysosomal homeostasis in dopaminergic neurons.

### Nurr1 modulates lysosomal protein expression through transcriptional regulation of GBA

**FIGURE 6:**
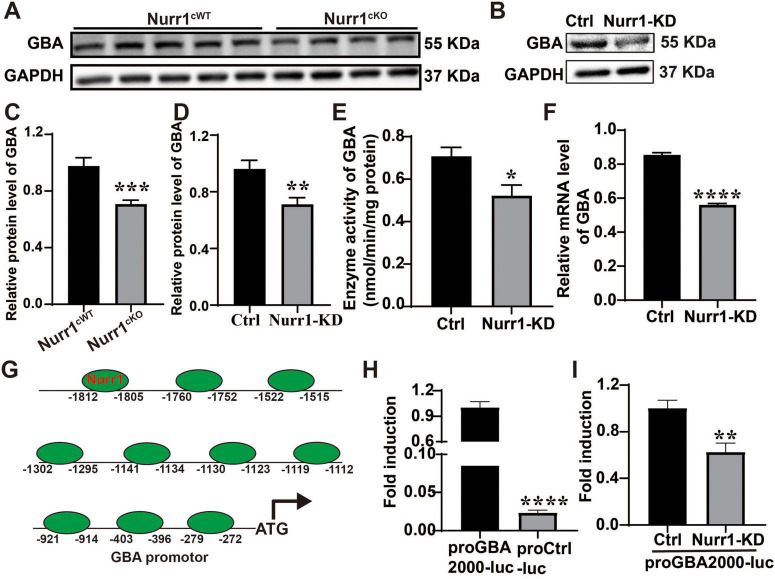
Nurr1 deficiency downregulated the expression and transcription of GBA. **(A)** Western blotting showed the expression of GBA in the SN of Nurr1^cKO^ and Nurr1^cWT^ mice. **(B)** The expression of GBA was indicated by Western blotting in Nurr1-KD and Ctrl cells. **(C,D)** Relative quantification of GBA expression *in vivo* and *in vitro*, normalized by GAPDH. **(E)** Enzyme activity of GBA. **(F)** The mRNA level of GBA was detected by RT-qPCR. **(G)** A schematic diagram of the binding sites of Nurr1 with GBA promoter (–2,000 to 0). **(H)** Successful construction of the plasmid containing GBA promoter (–2,000 to 0). **(I)** Transcriptional activity difference was confirmed by dual-luciferase reporter gene assay. Data were expressed as mean ± SEM. *p < 0.05 vs. Control, **p < 0.01 vs. Control, ***p < 0.001 vs. Control, ****p < 0.0001 vs. Control. Samples were collected at least three times with three replicates.

Western blotting analysis showed that knockdown of Nurr1 decreased the protein level of GBA to 74.0% and 74.0% compared to the control group *in vivo* and *in vitro*, respectively (*p* < 0.01, Figures 6A–D). As expected, lower GBA protein level led to a significant reduction of GCase activity, with 73.7% activity remaining (*p* < 0.05, Figure 6E). The mRNA level of GBA decreased to 65% in Nurr1-KD cells compared to Ctrl cells (*p* < 0.0001, Figure 6F). To investigate the molecular mechanisms underlying the regulation of GBA expression by Nurr1, we analyzed the potential binding sites of Nurr1 with the GBA promoter (−2,000 to 0) using JASPAR database. The results showed that the GBA promoter region contains 10 binding sites of Nurr1, suggesting that Nurr1 may be the transcription factor of GBA (Figure 6G). To confirm the hypothesis, we successfully constructed a luciferase plasmid containing the GBA promoter region ranged from −2,000 to 0 (*p* < 0.0001, Figure 6H). The dual-luciferase reporter gene assay showed that knockdown of Nurr1 significantly inhibited the transcriptional activity of GBA with an efficiency of 62.5% (*p* < 0.01, Figure 6I).

**FIGURE 7:**
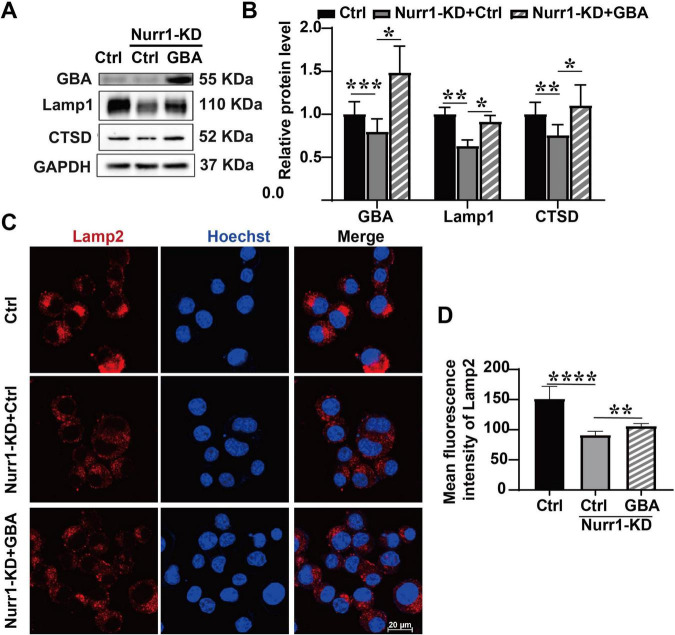
Impacts of GBA overexpression on the expression of lysosomal proteins. **(A)** Western blotting detection of GBA, Lamp1, and CTSD in Ctrl + Vector, Nurr1-KD + Vector, and Nurr1-KD + GBA group. **(B)** Relative quantification of these proteins, normalized by GAPDH. **(C)** Immunofluorescence staining of Lamp2 in the three groups. **(D)** Quantification of Lamp2 expression detected by immunofluorescence staining. Data were expressed as mean ± SEM. *p < 0.05, **p < 0.01, ***p < 0.001, ****p < 0.0001. Samples were collected at least three times with three replicates.

**FIGURE 8:**
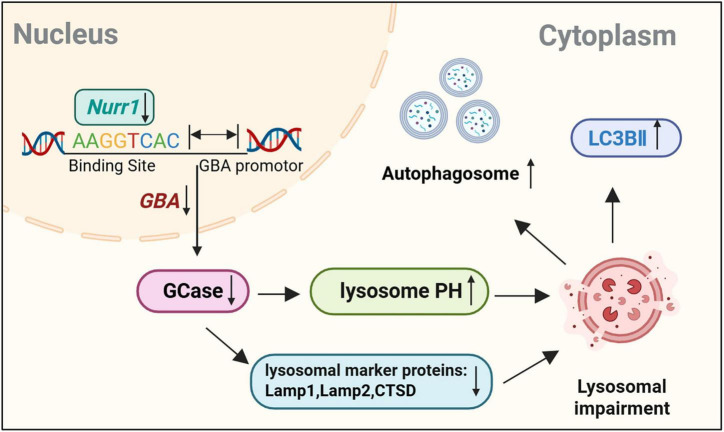
The graphical summary figure of this study. Nurr1 deficiency induced the aggregation of autophagosomes, increased the expression of LC3-II, increased lysosomal pH and declined the expression level of lysosomal marker proteins via GBA.

To further investigate the role of GBA in Nurr1-mediated regulation of lysosomal function, we overexpressed GBA in Nurr1-KD cells and detected the expression levels of lysosomal proteins. Overexpression of GBA alleviated the decreased expression of Lamp1 and CTSD proteins, and their expression increased to 1.45-fold and 1.46-fold compared to Nurr1-KD + Ctrl group, respectively (*p* < 0.05, Figures 7A, B). Immunofluorescence staining demonstrated that the expression level of Lamp2 was elevated to 1.17-fold by GBA overexpression (*p* < 0.01, Figures 7C, D). We conclude that the lower expression and enzymatic activity of GBA induce lysosomal depletion in Nurr1-KD cells (Figure 8), and the overexpression of GBA ameliorates the defects of lysosomal proteins.

## Discussion

Dysfunctional lysosomal activity and impaired ALP drive the accumulation of toxic proteins (e.g., α-synuclein) and contribute to PD (Chu et al., 2009). A meta-analysis identified 18 genes (GBA, SIPA1L2, TMEM163, LAMP3, VPS13C, SNCA, LRRK2, etc.) out of 26 genetic loci across the genome associated with PD modulate ALP, indicating the indispensable role of ALP in the pathogenesis of PD (Cuajungco and Kiselyov, 2017; Cai et al., 2022; Pang et al., 2022). However, the molecular mechanisms of the key PD associated regulatory genes modulate ALP remain unclear. Genetic and other studies in patients with PD have identified Nurr1 as a potential susceptibility gene for PD (Le et al., 2002; Le et al., 2008; Liu et al., 2012). Nurr1 promotes the neurogenesis of dopaminergic neurons and represses inflammatory factors in PD (Chen et al., 2018). Additionally, it has been reported that Nurr1 promotes autophagy in H_2_O_2_-induced cardiac stem cells (Shi et al., 2017). In this study, we identified 22 terms and 45 DEGs associated with ALP by transcriptome analysis. These DEGs were significantly enriched in autophagy assembly, phagosome acidification, autophagosome, cytoplasmic vesicle, protein binding, and macromolecular complex binding. However, the exact role and mechanism of Nurr1 in autophagy remain uncertain.

Autophagy occurs at basal levels to maintain cellular homeostasis under physiological conditions (Le et al., 2020). The LC3 protein exhibits a diffuse cytoplasmic distribution in its LC3-I form under basal conditions, and undergoes conversion to autophagosome membrane-associated LC3-II form, which appears as punctate aggregates during autophagy activation (Rajendran et al., 2024). Autophagosome formation is reflected by the LC3-II protein level and the intracellular LC3-positive puncta. The autophagic process is regulated by several molecular pathways involving the lysosomes and other organelles (Fleming et al., 2022).Various lysosomal functions depend on the acidification of the lysosome lumen, especially the maturation and activity of the hydrolytic enzymes, which influence the degradation rates of lysosomal inner substrates (Root et al., 2021; Navarro-Romero et al., 2022). The acidic environment is also indispensable for the modification of cargoes delivered to the lysosome including lipids, ions, and proteins (Perera and Zoncu, 2016). Mounting evidences have showed deficiency in key autophagic markers is sufficient to generate nigral DA neurons neurodegeneration (Friedman et al., 2012). Lamp1 and Lamp2 are widely used lysosomal markers for evaluating lysosomal function as their wide expression and critical functions (Xicoy et al., 2019). They participate partially in maintaining lysosomal integrity, pH, and catabolism (Eskelinen, 2006). CTSD is a lysosomal enzyme responsible for the degradation of α-synuclein and its insufficiency promotes the cell-to-cell transmission of α-synuclein aggregates (Bae et al., 2015). It is essential for maintaining lysosomal function, and neuronal lysosomes lacking CTSD are proteolytically inactive (Bae et al., 2015). It has been associated with severe phenotypes such as neurodegeneration in the brain, and reduced CTSD expression is closely related to PD pathology (Sevlever et al., 2008).

In an adeno-associated virus (AAV) vector-mediated overexpression of human wild-type α-syn rat model with PD-like neuropathology, reduced levels of Lamp1 (−11%) and CTSD (−33%) indicating lysosomal depletion were observed when cell loss occurred (Decressac et al., 2013a). CTSD was also reduced in the nigral neurons in PD patients, especially in neurons containing α-syn inclusions (Moran et al., 2007). An increased number of autophagosomes have been observed in cultured cells exposed to parkinsonian neurotoxins, as well as in the postmortem brain samples from PD patients (Mak et al., 2010). Deficiency of Lamp2 caused accumulation of autophagosomes in human cardiomyocytes (Chi et al., 2018). The isoform Lamp2a acts as the receptor to bind substrate proteins and then transfers them into the lysosome in chaperone-mediated autophagy (CMA). The oligomerization of Lamp2a affects CMA activity and lower levels of Lamp2a have been reported in PD models and samples from PD patients (Mak et al., 2010).

Nurr1 is critically involved in midbrain DA neuron function (Decressac et al., 2013b). Heterozygous Nurr1-deficient (*Nurr1*^+/–^) mice (older than 15 months) exhibit decreased rotarod performance and locomotor activities, suggesting a motor impairment analogous to parkinsonian symptoms (Jiang et al., 2005). A substantial increase of α-synuclein pS129 was detected in the DAergic neurons in lipopolysaccharide-treated Nurr1^Cd11bcre^ conditional knockout mice (Dong et al., 2020). TH is regulated by Nurr1 and plays a crucial role in the synthesis of DA (Al-Nusaif et al., 2022). Ablation of Nurr1 at age 5 weeks resulted in a more modest reduction of TH immunostaining and no apparent loss of cell bodies compared with ablation at birth or embryogenesis (Kadkhodaei et al., 2013). In the current study, ablation of Nurr1 was achieved by crossing Nurr1-floxed mice with Cre mice under the treatment of tamoxifen. A dramatical decreased expression of TH was detected in the SN, without prominent loss of DA neuron. We observed a remarkable reduction of HVA in the SN of Nurr1^cKO^ mice, in accord with DA metabolites change of PD. Decreased expression of the key lysosomal proteins Lamp1/2 and CTSD has been detected *in vivo* and *in vitro* with Nurr1 deficiency. We found that knockdown of Nurr1 induced intracellular aggregation of autophagosomes *in vitro*. The accumulation of undegraded autophagosomes occupies a large portion of the neuron and interferes with cellular functions, which may ultimately contribute to neuronal dysfunction and death (Zucca et al., 2018). LC3-II, a membrane-associated marker of autophagosome (Kabeya et al., 2000), is normally degraded by lysosomal proteases once the autophagosome fuses with a lysosome. Increased endogenous expression of LC3II and elevated colocalization of exogenous GFP-LC3B with Lamp1 were confirmed in this study, indicating the lysosomal mediated clearance of AP may be impaired. Lysosomal alkalization was indicated in Nurr1-KD cells verified by staining and flow cytometry. Meanwhile, the expression level of CTSD within the lysosomes decreased significantly in Nurr1-KD cells. Lysosome dysfunction has been implicated with increased lysosomal pH and deprived level of lysosomal marker proteins, which explains the aggregation of autophagosomes.

GCase encoded by GBA is one of the key acid hydrolases within the lysosome to help the degradation of molecules and organelles as well as the most common PD risk factor. An inverse correlation has been established between GCase activity and α-synuclein accumulation in GBA-mutant and sporadic PD brains (Gündner et al., 2019; Smith and Schapira, 2022). A combination of reduced protein levels and decreased catalytic activity of Gcase were identified in PD brains and most pronounced in the SN (Gegg et al., 2012). Decreased enzyme activity of GBA was detected in Nurr1-KD cells in coordination with reduced protein level *in vivo* and in intro. GBA was downregulated by Nurr1, which was predicted by transcriptome sequencing. This hypothesis was further confirmed by the markedly declined mRNA level of GBA when Nurr1 was knocked down. The relative activity assays showed that knockdown of Nurr1 significantly reduced the transcription activity of GBA. Evidence suggested a strong association between GBA and ALP regulation in experimental PD models (Ysselstein et al., 2019). The loss of GCase activity generates extensive lysosomal dysfunction, promoting the reduction of other representative lysosomal enzymes and intralysosomal pH changes, and affecting lysosomal membrane stability in human dopaminergic-like neuroblastoma BE(2)-M17 cells (Navarro-Romero et al., 2022). To clarify the relationship between GBA and lysosomal proteins in Nurr1-deficient model, GBA turnover assay was carried out to show that decrease of lysosomal markers can be alleviated by overexpression of GBA, further indicating the involvement of GBA in the regulation of lysosomal function in Nurr1-deficient model.

Our work demonstrated lysosomal and autophagy dysfunction in the Nurr1-dificient cell and mouse model, and Nurr1 participated in the regulation of the transcriptional activity of GBA (Figure 8). Nurr1 was involved in the ALP with declined GBA protein expression and enzymatic activity which indicated the interaction between PD-related genes, and the intensive mechanisms need to be further discussed.

## Data Availability

The transcriptome sequencing data are available at https://www.ncbi.nlm.nih.gov/sra/PRJNA1273283.
