# Supplementary material for: Nurr1 deficiency impairs autophagy-lysosomal function through GBA-dependent transcriptional regulation in Parkinson’s disease pathogenesis
Source: Front Aging Neurosci. 2025 Jun 30;17:1612389. doi: 10.3389/fnagi.2025.1612389 (PMC12256511; doi:10.3389/fnagi.2025.1612389)
Supplement: Supplementary file 1 [file Data_Sheet_1.pdf]

the original images of Western blot

Fig 1B

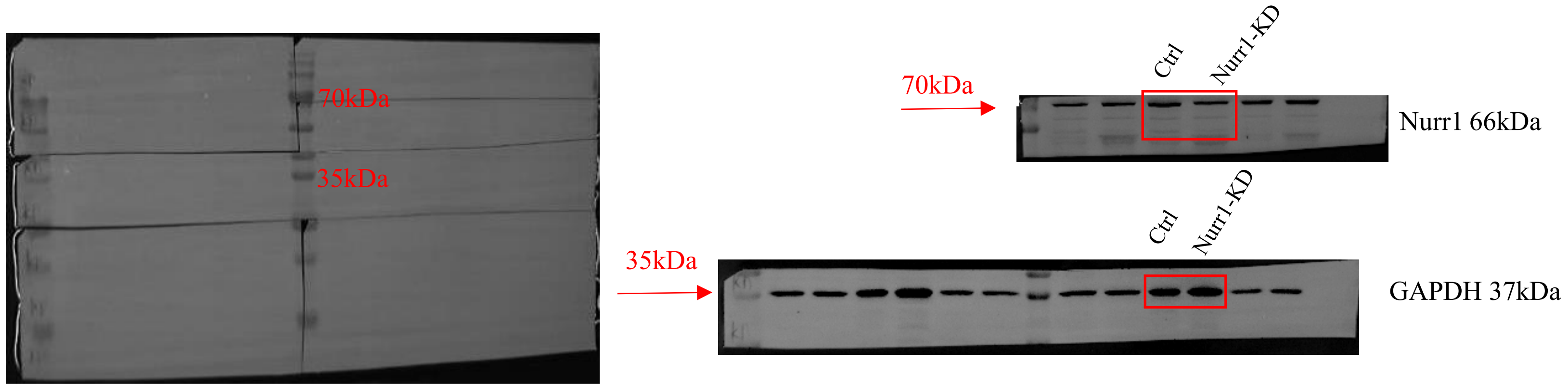

Fig 3G

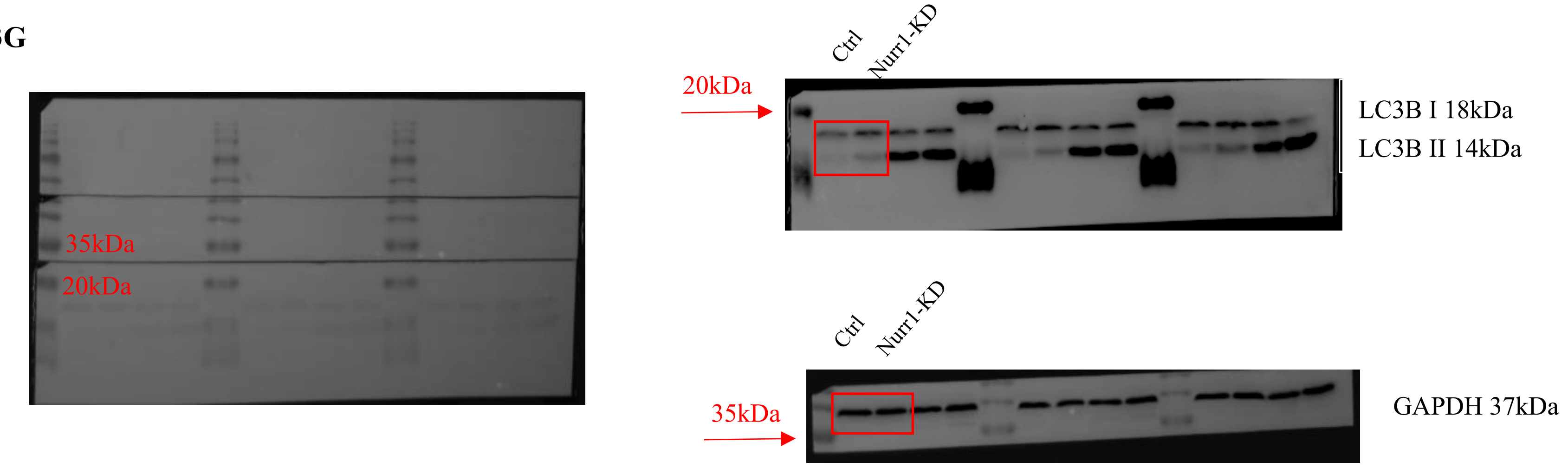

Fig 4D

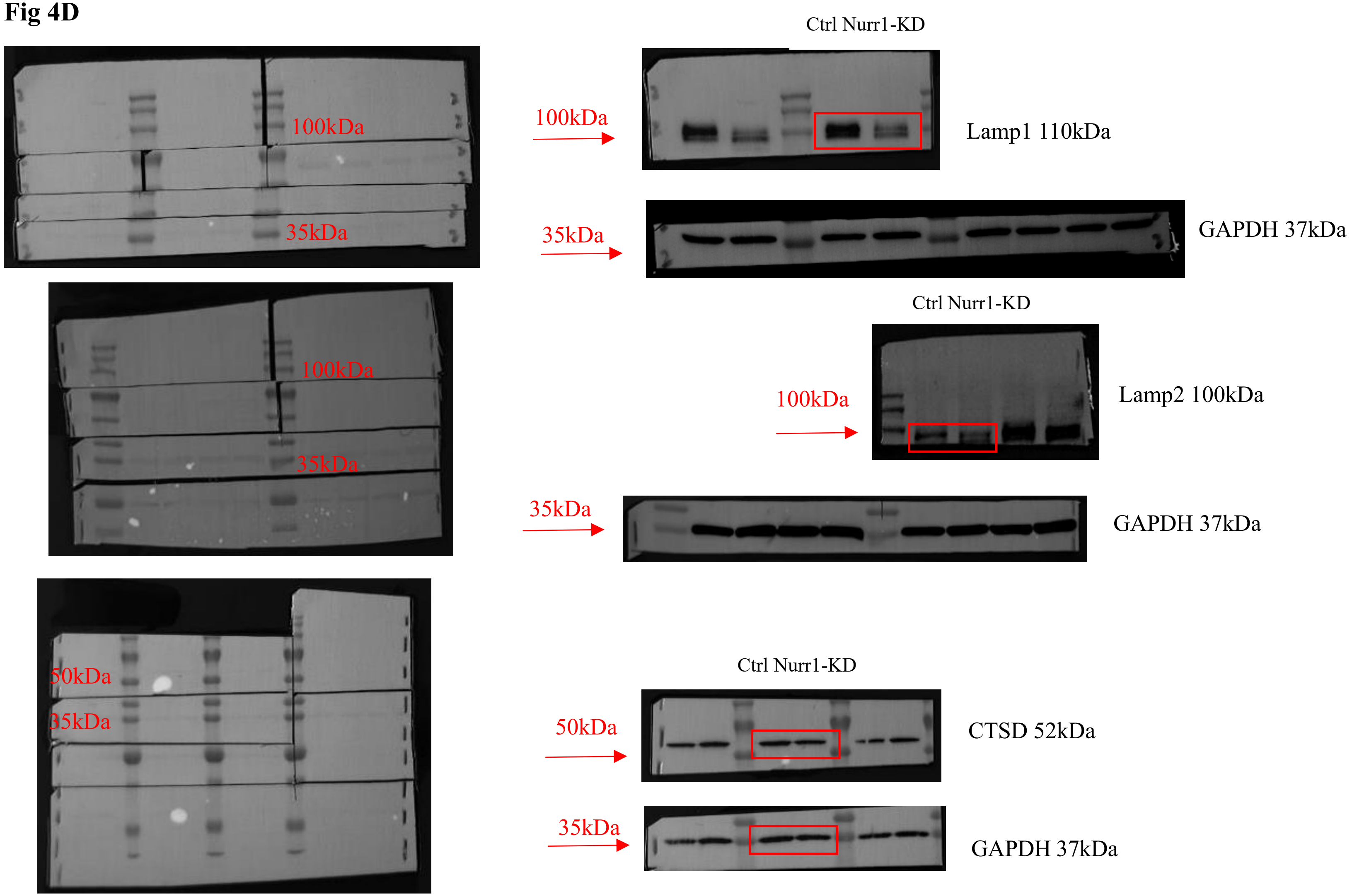
100kDa
35kDa
100kDa
35kDa
50kDa
35kDa
Ctrl Nurr1-KD
100kDa
Lamp1 110kDa
35kDa
GAPDH 37kDa
Ctrl Nurr1-KD
100kDa
Lamp2 100kDa
35kDa
GAPDH 37kDa
Ctrl Nurr1-KD
50kDa
CTSD 52kDa
35kDa
GAPDH 37kDa

Fig 5E

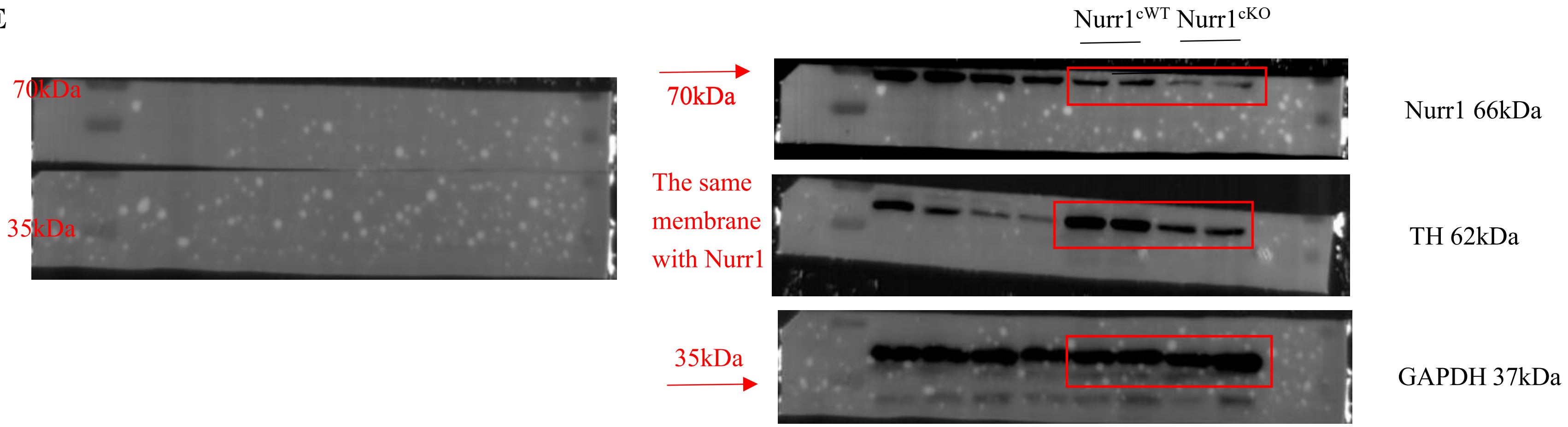
70kDa
35kDa
Nurr1cWT Nurr1cKO
70kDa
The same membrane with Nurr1
35kDa
Nurr1 66kDa
TH 62kDa
GAPDH 37kDa

Fig5I

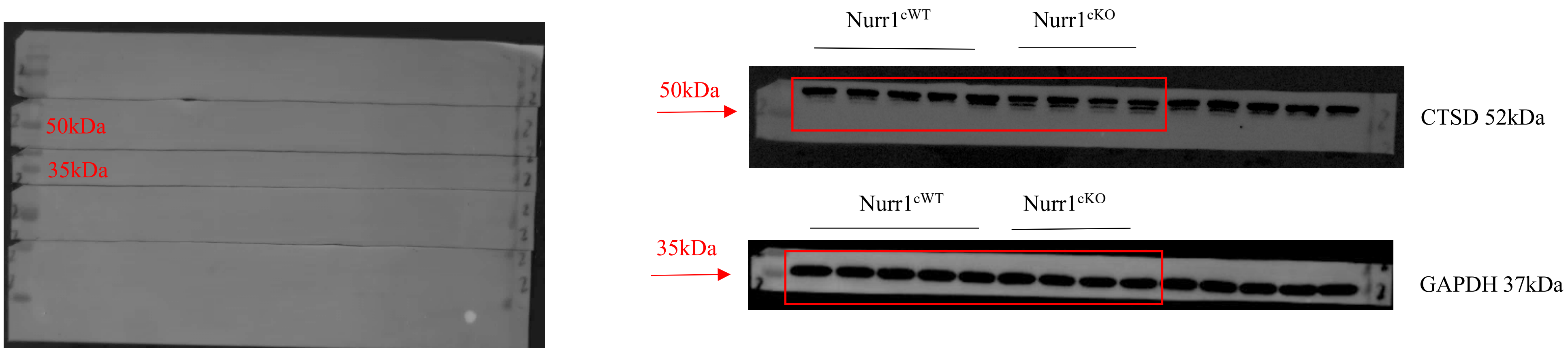
50kDa
35kDa
Nurr1cWT
Nurr1cKO
50kDa
CTSD 52kDa
Nurr1cWT
Nurr1cKO
35kDa
GAPDH 37kDa

Fig5J

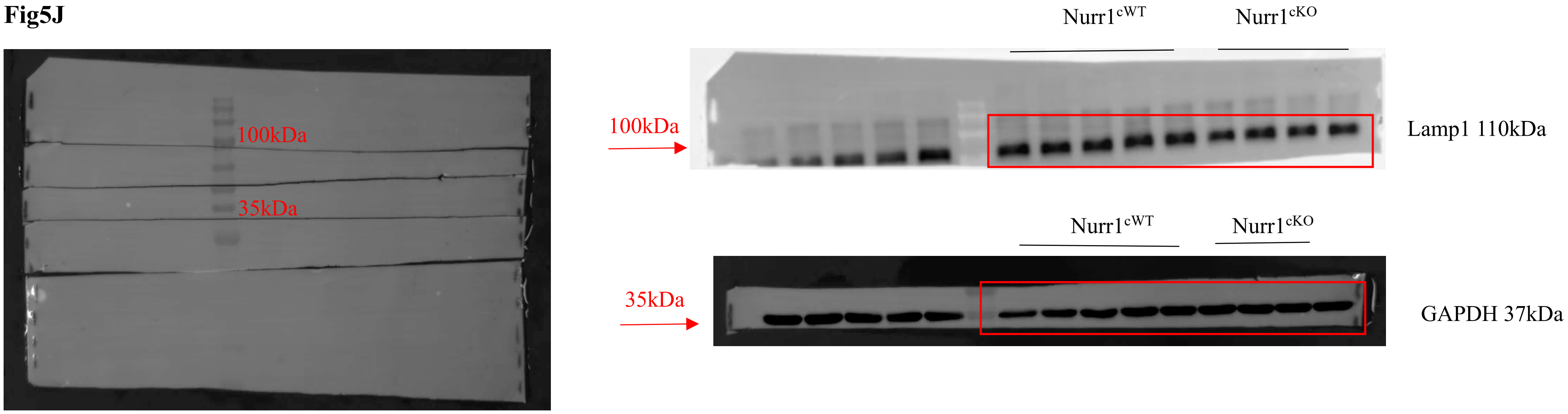

Fig 6A

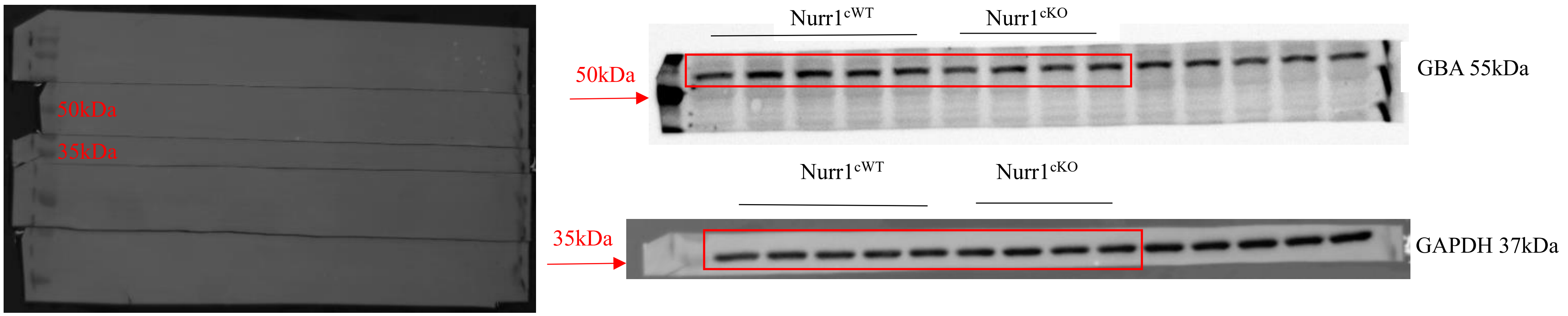
50kDa
35kDa
Nurr1cWT
Nurr1cKO
50kDa
GBA 55kDa
Nurr1cWT
Nurr1cKO
35kDa
GAPDH 37kDa

Fig 6B

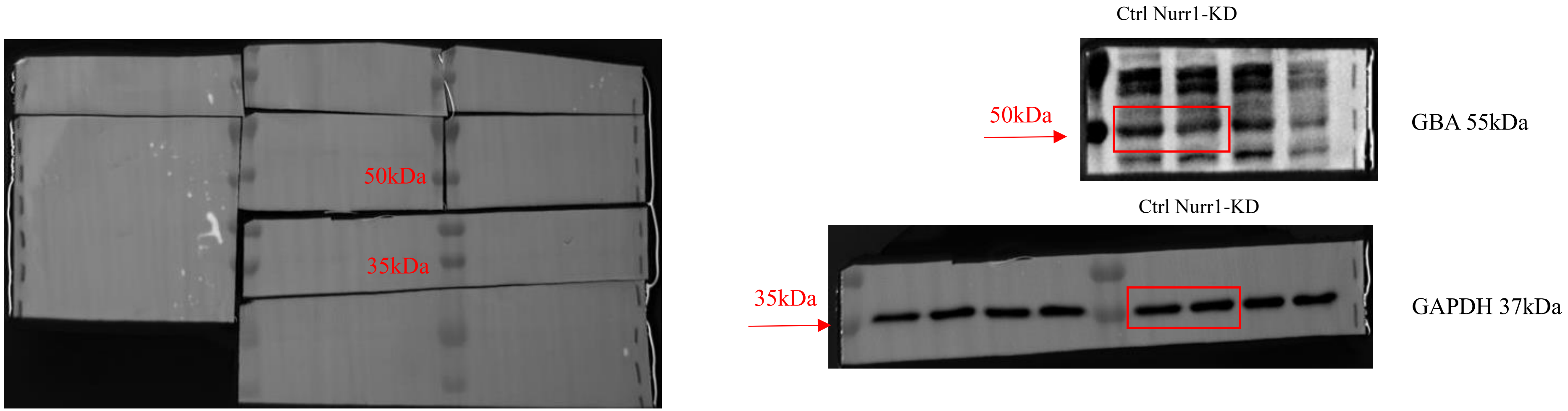
50kDa
35kDa
Ctrl Nurr1-KD
50kDa
GBA 55kDa
Ctrl Nurr1-KD
35kDa
GAPDH 37kDa

Fig 7A

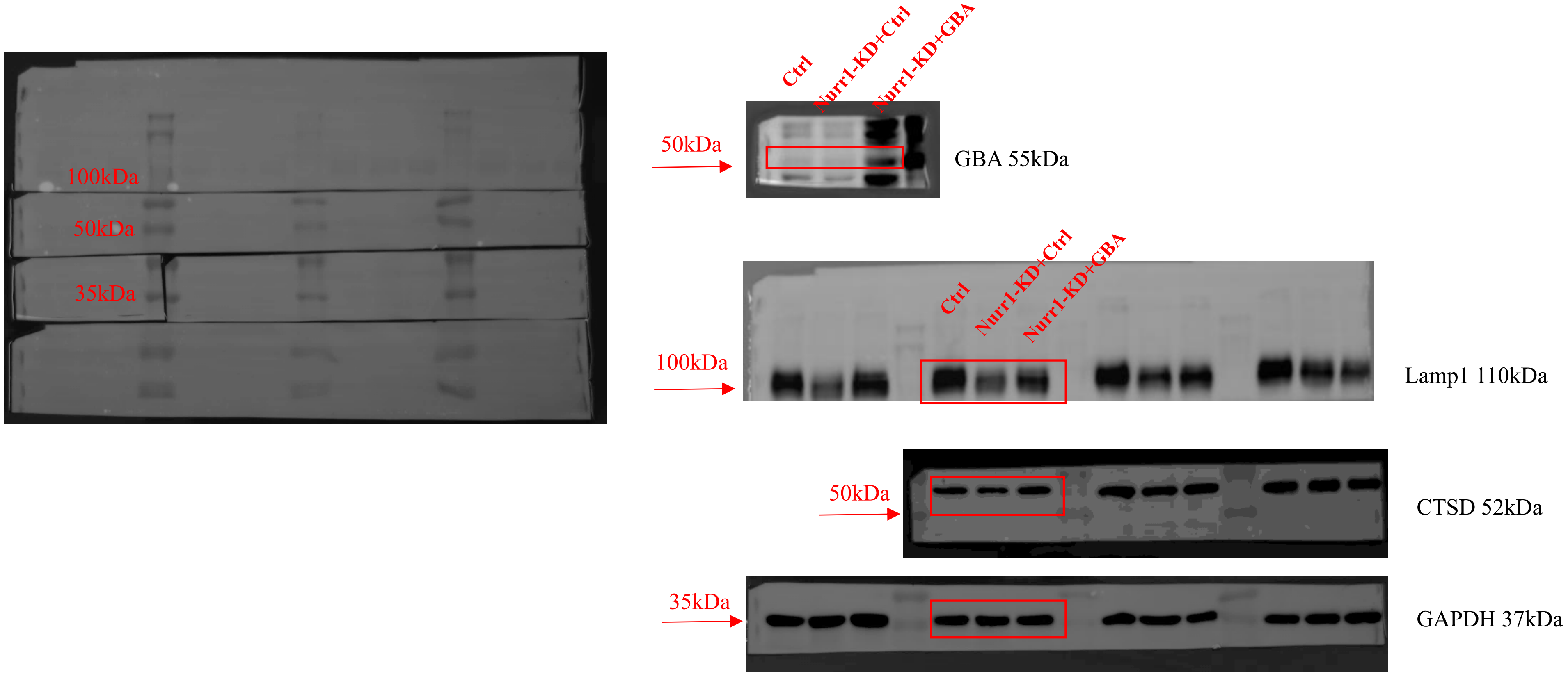
100kDa
50kDa
35kDa
Ctrl
Nurr1-KD+Ctrl
Nurr1-KD+GBA
50kDa
GBA 55kDa
Ctrl
Nurr1-KD+Ctrl
Nurr1-KD+GBA
100kDa
Lamp1 110kDa
50kDa
CTSD 52kDa
35kDa
GAPDH 37kDa
